# Cell-Specific Post-Transcriptional Regulation of γ-Synuclein Gene by Micro-RNAs

**DOI:** 10.1371/journal.pone.0073786

**Published:** 2013-09-11

**Authors:** Irina Surgucheva, Sumedha Gunewardena, H. Shanker Rao, Andrei Surguchov

**Affiliations:** 1 Retinal Biology Research Laboratory, Veterans Administration Medical Center, Kansas City, Missouri, United States of America; 2 Department of Neurology, Kansas University Medical Center, Kansas City, Kansas, United States of America; 3 Department of Molecular and Integrative Physiology, Kansas University Medical Center, Kansas City, Kansas, United States of America; Bascom Palmer Eye Institute, University of Miami School of Medicine, United States of America

## Abstract

γ-Synuclein is a member of the synucleins family of small proteins, which consists of three members:α, β- and γ-synuclein. γ-Synuclein is abnormally expressed in a high percentage of advanced and metastatic tumors, but not in normal or benign tissues. Furthermore, γ-synuclein expression is strongly correlated with disease progression, and can stimulate proliferation, induce invasion and metastasis of cancer cells. γ-Synuclein transcription is regulated basically through the binding of AP-1 to specific sequences in intron 1. Here we show that γ-synuclein expression may be also regulated by micro RNAs (miRs) on post-transcriptional level. According to prediction by several methods, the 3′-untranslated region (UTR) of γ-synuclein gene contains targets for miRs. Insertion of γ-synuclein 3′-UTR downstream of the reporter luciferase (LUC) gene causes a 51% reduction of LUC activity after transfection into SKBR3 and Y79 cells, confirming the presence of efficient targets for miRs in this fragment. Expression of miR-4437 and miR-4674 for which putative targets in 3′-UTR were predicted caused a 61.2% and 60.1% reduction of endogenous γ-synuclein expression confirming their role in gene expression regulation. On the other hand, in cells overexpressing γ-synuclein no significant effect of miRs on γ-synuclein expression was found suggesting that miRs exert their regulatory effect only at low or moderate, but not at high level of γ-synuclein expression. Elevated level of γ-synuclein differentially changes the level of several miRs expression, upregulating the level of some miRs and downregulating the level of others. Three miRs upregulated as a result of γ-synuclein overexpression, i.e., miR-885-3p, miR-138 and miR-497 have putative targets in 3′-UTR of the γ-synuclein gene. Some of miRs differentially regulated by γ-synuclein may modulate signaling pathways and cancer related gene expression. This study demonstrates that miRs might provide cell-specific regulation of γ-synuclein expression and set the stage to further evaluate their role in pathophysiological processes.

## Introduction

γ**-**Synuclein was first identified by a differential cDNA sequencing approach and was termed the breast-cancer-specific gene 1 (BCSG1). The gene is expressed in high abundance in a breast cancer (BC) cDNA library but scarcely in a normal breast cDNA library [1]. Later the overexpression of γ-synuclein has been shown in several types of cancer [2]–[6]. In cancer cells γ-synuclein significantly increases the ligand-dependent transcriptional activity of estrogen receptor-alpha (ER-alpha). Overexpression of γ-synuclein stimulates the ligand-dependent cell proliferation and estrogen-induced activation of ERK1/2, whereas suppression of endogenous γ-synuclein expression considerably inhibits cell growth in response to estrogen. Furthermore, γ-synuclein functions as a molecular chaperone protein for membrane-bound estrogen receptor (ER)-alpha [7], [8]. Although highly elevated level of γ-synuclein is documented in many types of cancer, the triggers initiating its high expression as well as the downstream molecular events of such upregulation are poorly understood. In addition to cancer, deregulation of γ-synuclein accompanied by the formation of aberrant inclusions is also associated with neurodegenerative diseases [9]–[13].

γ-Synuclein is a member of the synuclein family consisting of α,- β, and γ-synucleins [2], [6], [13], [14]. Another member of the same family, α-synuclein is the most thoroughly studied member of the family, because of its proven association with the Parkinson’s disease and other neurodegenerative disorders [14]–[17].

One of the regulatory mechanisms of the γ-synuclein tissue-specific expression is realized through the binding of activator protein AP-1 to *cis-*regulatory sequences localized in the intron 1 of the gene. γ-Synuclein expression is also regulated by methylation of the nucleotide sequence of the CpG island [18]–[20]. The mechanism of AP-1 dependent transcriptional regulation of γ-synuclein expression is different in glial and neuronal cells and is altered in cancer cell line [4], [18]–[21].

In spite of an established role of the deregulation of γ-synuclein expression in cancer and other human diseases the mechanism of its post-transcriptional regulation is not investigated. Modulation of gene expression through the binding of small micro-RNAs (miRs) to the 3′-untranslated region is currently proven for many genes. The defects of this mechanism are associated with many diseases, including malignancy and neurodegeneration [22], [23]. Recent studies point to the existence of collaborative regulation of gene expression by transcription factors and miRNAs [23] in miRs targetome networks.

The expression of another member of the synuclein family, α-synuclein is regulated by binding of miRs to the 3′-untranslated region (3′-UTR) of its mRNA. This type of regulation plays an essential role in the transition of normal to pathogenic functions of α-synuclein [24]–[28]. At least two miRs, miR-7 and miR-153 are involved in 3′-UTR mediated negative control of α-synuclein expression [27]–[30]. Furthermore, miR-133b is deficient in the Parkinson’s disease midbrain, and miR-34b/34c is decreased in certain brain regions in patients [24].

miRs are small non-coding RNAs that cause the translational repression or cleavage of target messages. Mature miRs repress protein expression primarily through base pairing of a seed region with the 3′-UTR of the target, thus regulating gene expression in various cell types [22], [23], [31]–[34]. There is a growing list of miR genes that play specific roles in various human cancers, affecting cancer predisposition, initiation and progression, as well as suppressing or enhancing metastasis, etc. Some miRs demonstrate oncogenic properties, whereas the others play a role of tumor suppressor. Due to these features miRs are considered as newer therapeutic targets [32], [35]. Furthermore, because of the widespread deregulation of miRs in all types of tumors, their expression profiles are useful diagnostic and prognostic signature classifiers. Understanding of mechanisms regulating γ-synuclein expression is important because of its role in the development of devastating human diseases.

In the current study we have examined a role of γ-synuclein 3′-UTR and miRs in post-transcriptional regulation of its expression. In addition, we studied how upregulation of the γ-synuclein gene affects pattern of the miRs expression and modulates downstream pathways. The results indicate that miRs are essential cell-specific regulatory elements which modulate γ-synuclein expression on posttranscriptional level in addition to previously described mechanisms of transcriptional regulation. Interestingly, this mechanism is efficient only at low or moderate level of expression, but not in cells overexpressing γ-synuclein. We also present data on downstream effects of elevated γ-synuclein showing how it alters miRs and signaling pathways. These regulatory mechanisms might play an important role in spreading the pathology. We discuss how these epigenetic mechanisms contribute to the role of γ-synuclein in tumor development and progression of other pathology.

## Methods

### Cell Line Cultures and Transfection

The following cell lines have been used.

1. Human neuroblastoma cell line SH-SY5Y (ATCC cat. number CRL-2266™) derived from metastatic bone marrow [36]. These cells grow in DMEM (Gibco, Cat. # 11965-092), containing 10% FBS (Equitech-Bio, Inc, Cat#SFBM), Sodium Pyruvate (Gibco, 1136–070) and Antibiotic-Antimycotic (Gibco, Cat. # 152-40-062, dilution 1∶100). The cells were split every 3–4 days.
2. SH-SY5Y clone overexpressing γ-synuclein (stable B9 clone) was generated after transfection of the parent SH-SY5Y by pcI-Neo-gamma synuclein DNA construct. These cells were grown in the same media in the presence of 400 µg/ml of antibiotic G-418.
3. Human breast cancer cell line SK-BR-3 (ATCC Cat. Number HTB-30™) is derived from metastatic pleural effusion due to an adenocarcinoma originating in the breast. The cells were grown in McCoy’s 5a Media (Cellgro, Cat. #10-050-CV), containing 10% FBS (Equitech-Bio Inc., Cat # SFBM) and Antibiotic-Antimycotic in 1∶100 dilution (Gibco, Cat. # 152-40-062).
4. Retinoblastoma cell line Y79 (ATCC Number HTB-18™) were grown in RPMI-1640 Medium (Cellgro, Cat. # 10-041-CV), containing 20% FBS (Equitech-Bio, Inc, Cat # SFBM) and Antibiotic-Antimycotic.

### Generation of DNA Constructs

#### γ-Synuclein ID information - HGNC:11141

3′-UTR of γ-synuclein gene was amplified by PCR using pcI-Neo plasmid with inserted γ-synuclein cDNA used as a template. Primers contained restriction sites at the ends (*Xho I* at 5′-end and *Not I* at the 3′-end). After purification the PCR-product was inserted into psiCHECK-2 vector (Promega).

### Luciferase (LUC) Reporter Assay

**Figure 1:**
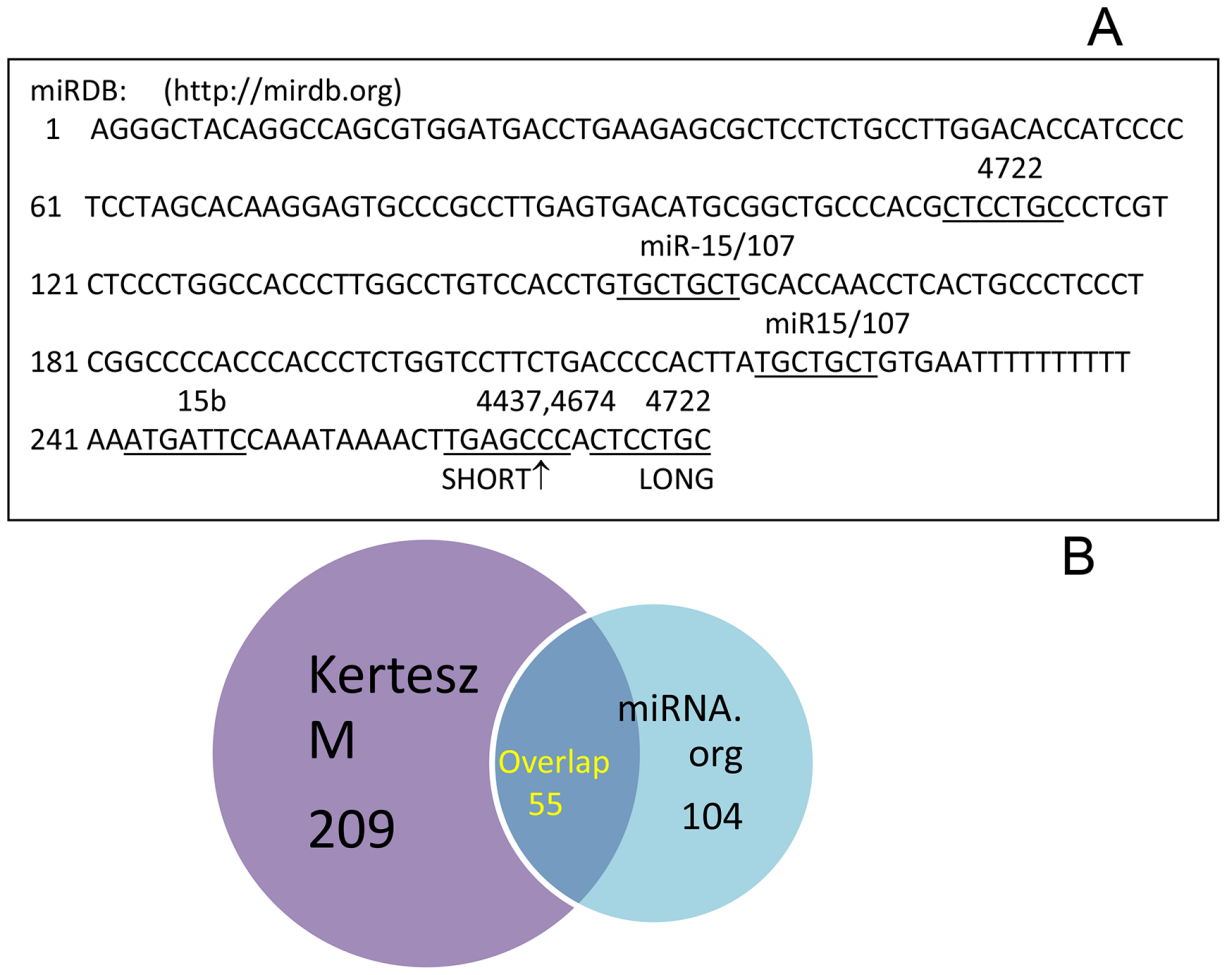
Nucleotide sequence of γ-synuclein 3′-UTR and prediction of miRNAs targets. **A**. γ-Synuclein 3′-UTR. Arrows shows a short form of the UTR (UTR-S). The short form does not contain complete target for miR-4437 and miR-4674. Putative targets for miRs are shown above the sequence and the corresponding sequences are underlined. **B**. Venn diagram of two miR target prediction methods.

Two PCR products corresponding to the 3′-UTR of γ-synuclein were generated: a 275 bp (long form) and 265 bp (short form) as shown on **Figure 1A**. We also used truncated forms of the long form from which some of the putative miRs targets were deleted. All generated forms of the 3′-UTR were inserted in a Luc reporter vector pSICHECK2 (Promega) and tested for Luc activity after transient transfection.

psiCHECK2 vectors contain a multiple cloning region downstream of the stop codon of an SV40 promoter-driven Renilla LUC gene ensuring the expression of a Renilla transcript with the 3′UTR sequence of interest. Renilla luciferase activity is used to assess the effect of the 3′UTR on transcript stability and translation efficiency. The psiCHECK-2 vector also contains a constitutively expressed firefly LUC gene. Firefly luciferase is used to normalize transfection and eliminate the need to transfect a second control vector.

For transfection and determination of Luc-activity the cells were split into 24 or 12-well plates in the complete media. Lipofectamine 2000 (Life Technology), 500 ng of plasmid DNA and 2.5 µl of the transfection agent were used for 24 well plate. After 24 h or 48 h the LUC activity was measured by a Dual Luciferase Reporter assay System 10-Pack (Promega, Madison, WI, E-1960) as recommended by the manufacturer. The Synergy HT Multi-Detection Microplate Reader was used for the detection of luciferase activity as described earlier [21], [37]. The ratio between the luminescence of Renila and Firefly in control vector was taken as 100%. The luminescence of each sample was measured in triplicate and an average was taken for further calculations.

Deletion of putative targets for the mir-103 or mir-107 from 3′-UTR was carried out using the QuikChange Lightning Site-Directed Mutagenesis Kit (Agilent Technologies, Cat. # 210519). The sequences of primers used for the deletions are presented in Table S2. All DNA constructs were verified by sequencing.

The sequences of primers used for the insertion of L and S forms of γ-synuclein 3′-UTR into the vector and for the expression of miRs using “BLOCK-iT Pol II miR RNAi Expression Vector Kit are presented in the Table S2.

### Construction of a Vector Expressing miRNA

For generation of a vector expressing miRNA two single–stranded DNA oligonucleotides were designed: one encoding the target pre-miRNA (“top-strand” oligo) and the other its complement (“bottom strand” oligo) as described above. Each designed miRNA mimic was 64 nucleotides in length, including partial flanking sequences, mature miRNA sequences, terminal loop and sense target sequences. Using the BLOCK-iT Pol II miR RNAi expression vector kit (Invitrogen, CA), the oligos were annealed and the engineered pre-miRNAs were cloned into the cloning site (ACGA and CAGG) of pcDNA 6.2-GW/EmGFP-miR vectors. These sites were flanked on either side to allow directional cloning or proper processing of pre-miRNA. Each plasmid was sequenced to confirm the inserted double stranded miRNAs oligos, using EmGFP forward sequencing primer. The expressing plasmids were electroporated into corresponding cells which were analyzed for miRs expression. Ligation reaction, transformation and subsequent steps were performed as recommended by the manufacturer.

### miRs Isolation

miRs were isolated using “mirVana miRNA isolation kit from Ambion.10^5^–10^6^ cultured mammalian cells were washed with cold PBS and lysed in the Lysis/Binding solution. 1/10 volume of the homogenate additive was added to the lysate for 10 min and the mixture was treated with an equal volume of acid-phenol: chlorophorm. Aqueous layer was absorbed on the filter and treated with ethanol as recommended by the manufacturer. The samples were DNase digested and the quality of miRs was determined by electrophoresis in denaturing acrylamide gel (1% agarose −2% formaldehyde). The ration of A260:A280 was at least 1.95.

### qRT-PCR

Amplification was performed on an Applied Biosystems 7300 instrument using the following conditions: 2 min at 50°C (hold), 10 min at 95°C (hold), 15 s at 95°C, and 1 min at 60°C (45 cycles). The samples were amplified in triplicate and three independent isolations of RNA/cDNA were used. In each experiment non-template controls (without cDNA) did not give amplification signals.

### MicroRNA Microarrays

RNA samples were analyzed by Ocean Ridge Biosciences (ORB, Palm Beach Gardens, FL) using custom multi-species microarrays containing 1718 probes covering 1733 human mature microRNAs present in the miRBase version 17.0 database. The sensitivity of the microarray is such that it could detect as low as 20 amoles of synthetic microRNA being hybridized along with each sample. The microarrays were produced by Microarrays Inc. (Huntsville, Alabama), and consisted of epoxide glass substrates that have been spotted in triplicate with each probe. The microarrays analysis was performed as follows (A-D):

**A**. Sample Processing. Quality of the total RNA samples was assessed using UV spectrophotometry and agarose gel electrophoresis. The samples were DNAse digested and low-molecular weight (LMW) RNA was isolated by ultrafiltration through Nanosep 100 K columns (Pall Corporation) and subsequent purification using the RNeasy MinElute Clean-Up Kit (Qiagen). The LMW RNA samples were 3′-end labeled with Oyster-550 fluorescent dye using the Flash Tag RNA labeling Kit (Genisphere). Labeled LMW RNA samples were hybridized to the MicroRNA microarrays according to conditions recommended in the Flash Tag RNA labeling Kit manual. The microarrays were scanned on an Axon Genepix 4000B scanner, and data was extracted from images using GenePix V4.1 software.

**B**. Data Pre-processing. Spot intensities were obtained for the 8816 features on each microarray by subtracting the median local background from the median local foreground for each spot. The log2-transformed spot intensities for all 8816 features were normalized, by subtracting N from each spot intensity, and scaled by adding the grand mean of N across all microarrays. The mean probe intensities for each of the human probes on each array were then determined by averaging the triplicate spot intensities. The 1718 human non-control log2-transformed, normalized, and averaged probe intensities were filtered to obtain a list of 821 human microRNA probes showing probe intensity above T in all samples from at least one treatment group.

**C**. Microarray Quality Control. Each array contains probes targeting 11 different synthetic miRNAs, each of which is added at a mass of 200 amoles to each RNA sample prior to labeling and hybridization. Sensitivity of the microarray hybridization was confirmed by detection of hybridization signal for all 11 spikes well above the detection threshold. The array also contains a set of specificity control probes complementary to three different miRNAs. Each specificity control includes a perfect match, single mismatch, double mismatch, and shuffled version of the probe. Specificity of the hybridization was confirmed by detection of hybridization signal on the microarray for the perfect match probes and not the double mismatch and shuffled version of the probes. Reproducibility of the arrays was determined by monitoring the hybridization intensity for the triplicate human spots on each array. The sensitivity, specificity, and reproducibility data for the arrays were compiled into a Quality Control report.

**D**. Differential Expression Analysis. For statistical analysis, samples were binned into four treatment groups (B9, SHSY5Y, SKBR3, and Y79). The log2-transformed and normalized probe intensities for the 821 non-saturated and detectable human probes were examined for differences between all four groups by 1-way ANOVA using National Institute of Ageing (NIA) Array Analysis software [38]. This ANOVA was conducted using the Bayesian Error Model and 20 degrees of freedom. A total of 461 probes showed significant differences with FDR <0.1. The statistical significance was determined using the False Discovery Rate (FDR) method which was proposed by Benjamini and Hochberg [39]. It is the proportion of false positives among all probes with P values lower or equal to the P value of the probes that we consider significant. It can also be viewed as an equivalent of a P-value in experiments with multiple hypotheses testing. FDR is an intermediate method between the P-value and Bonferroni correction (multiplying P-value by the total number of probes). In addition to ANOVA, Principal Component Analysis (PCA) was also performed on the 821 detectable human probe intensities using the module built in to the National Institute of Ageing (NIA) Array Analysis software.

**E**. Hierarchical Clustering of MicroRNA Array Data.

Data for the 461 detectable and significant human probes were clustered using Cluster 3.0 software [40). Genes were median centered prior to hierarchical clustering. Hierarchical clustering was conducted using Centered Correlation as the similarity metric and Average Linkage as the clustering method. Intensity scale shown is arbitrary.

**F**. Validation of microRNA microarray results.

The results of microRNA microarray were validated for selected miRs by qRT-PCR with miR-specific primers. A reverse transcription reaction using 250 ng of total RNA, was done using the QIAGEN miScript II RT kit (QIAGEN, Cat # 218161). cDNA Samples were assayed using a custom miRNA PCR array from QIAGEN. The array contained 14 assays for miRNAs (hsa-miR-199b-5p,hsa-miR-375,hsa-miR-10b-5p,hsa-miR-328,hsa-miR-532-5p,hsa-miR-660-5p,hsa-miR-497-5p, hsa-miR-143-5p, hsa-miR-183-5p, hsa-miR-885-5p, hsa-miR-221-5p, hsa-miR-204-5p, hsa-miR-146a-5p, and RNU6-2) and two assays for normalization (SNORD68 and SNORD96A). PCR arrays were done according to manufacture instructions using miScript SYBR Green PCR Kit (Cat # 218073). Real time PCR was done using an ABI 7900 SDS Real Time instrument (Life Technologies). Raw data was exported from the real-time instrument software and fold regulation was calculated using the Delta Delta CT method by the miScript miRNA PCR Array Data Analysis tool as described in http://www.sabiosciences.com/mirnaArrayDataAnalysis.php.

### Western Blotting

The details of Western blotting are described previously [11], [21]. Antibody to γ-synuclein was purchased from Abcam (Cat # ab55424), antibody to actin was from Chemicon MAB1501.

### Prediction of miR Targets in γ-synuclein 3′-UTR

The following software or algorithms were used for the prediction of miR targets: Target Scan, miRanda, Diana microT, Kertesz algorithm [41], miRDB and miRNA.org.

### Heat Map Generation

The heat-map of the miRNA expression profile in SH-SY5Y and stable B9 clones was generated by two-dimensional hierarchical clustering. Expression values of each miRNA were divided by the mean expression over all conditions and log transformed before clustering. The Ward’s method was used for the linkage function and applied on an Euclidean distance matrix of the pair-wise distances of the log transformed miRNA expression data. The color gradient of the heat-map represents the log of the relative expression of miRNAs to their mean expression over all conditions with red indicating over expression and green indicating under expression.

### Schema Illustrating the Effect of γ-synuclein Overexpression on miRs and Signaling Pathways

The network of molecular interactions associated with γ-synuclein and miRNAs was generated using Ingenuity Pathways Analysis (IPA, Ingenuity Systems, fall 2012 (www.ingenuity.com). This software allows to characterize potential molecular interactions associated with those molecules that are significantly altered by overexpression of γ-synuclein (i.e. molecules in **Table 1**). The software identifies significant interaction networks and biological functions in a set of molecules based on information gathered in the Ingenuity Pathway Knowledge Base (IPKB). The IPKB is a repository of information about genes and gene products that interact with each other. Once the network is generated, IPA assigns biological functions that are significantly associated with the molecules in the network where the significance is calculated by the right-tailed Fisher’s Exact test. This test basically measures the significance of the overlap between the molecules in the network and the molecules associated with a particular biological function.

**Table 1:** γ-Synuclein overexpression alters the level of several miRs.

	fold change					
	micro		qRT		Involvement in cancer	
miR	array		PCR	Ref		miRNA features
**Up-regulated miRs**						
mir-199b-5p		5.27	2.86	59		Medulloblastoma miR-199b-5p expression
						correlates with metastasis spread
mir-375		3.68		51	BC↑	Significantly upregulated in BC cells
mir-10b		3.07	1.3	49	BC↑	Promotes BC cell motility and invasiveness via
						a Rho-GTPase
				48	BC	Promotes metastasis
				68	BC↑	Upregulated in BC
				57	BC	Associated with tumor metastasis
mir-328		2.92	1.44	69	PC↑	Elevated in PC patients serum
mir-532-5p		2.77		70	melanoma↑	Up-regulated in melanoma lines and metastatic
						melanoma tumors
mir-660		2.39		71	leukemia	Increased in leukemic cells after HNE treatment
mir-138*		2.19		72	BC↑	Upregulated in BC
mir-497* +		2.17		73	BC↑	Upregulated in BC patients
mir-143		2.1		74		Targets DNA methyltransferases
mir-183		2.08		53		Oncogenic role in bladder cancer
/96/182						
				52		Upregulated in patients with lung cancer
mir-885-5p*		2.06	2.47	69	PC↑	Elevated in PC patients serum
				75		Activated p53 and inhibits proliferation and survival
miR-103+		1.57		45		Expression is a cancer prognostic biomarker
**Down-regulated miRs**						
mir-221	0.03			76	PC, other types	Downregulated in PC, aberrant regulation in
						other cancers
				77	multiple cancers	Modulate cancer by affecting multiple oncogenic
						pathways
mir-204	0.015		−1.6	54	Tumor suppressor	Lost in multiple cancers, including BC
mir-146a	0.27		−3.2	55	BC	Inhibits migration and invasion of cancer cells
				78	BC	Suppresses metastasis
mir-1268*	0.39			79	BC↓	Downregulated in BC
mir-125b	0.45			68	BC↓	Tumor suppressor, reduced in BC, affects HER2/3
				65	BC↓	Downregulated in BC. Suppresses oncogenes
						ERBB2/3

* for these miRs targets are predicted in γ-synuclein 3′-UTR (**Table 2**).

+ members of the 15/107 family of miRs.

### Activation z-score

The activation z-score is calculated by IPA and reflects the significance of the overlap of molecules in a data-set and their direction of expression to the molecules and their likely direction of expression (established from literature) associated with a biological function. The z-score predicts the direction of change for the function. An absolute z-score of ≥2 is considered significant. A function is Increased if the z-score is ≥2 and Decreased if the z-score ≤2.

### Statistical Analysis

At least three sets of transfection were performed for each experiment. A paired Student’s t test was used to assess significant differences between groups. For microRNA microarrays the 95th percentile of the negative control spots was calculated for each array. The spot intensities and 95th percentile of negative controls (TPT95) were transformed by taking the log (base 2) of each value. The normalization factor (N) for each microarray was determined by obtaining the 20% trim mean of the probes intensities above threshold in all samples. Other details for Microarrays statistical analysis are described above in “MicroRNA Microarrays”, D.

## Results

### Multiple miR Target Sites are Predicted in γ-synuclein 3′-UTR

**Figure 2:**
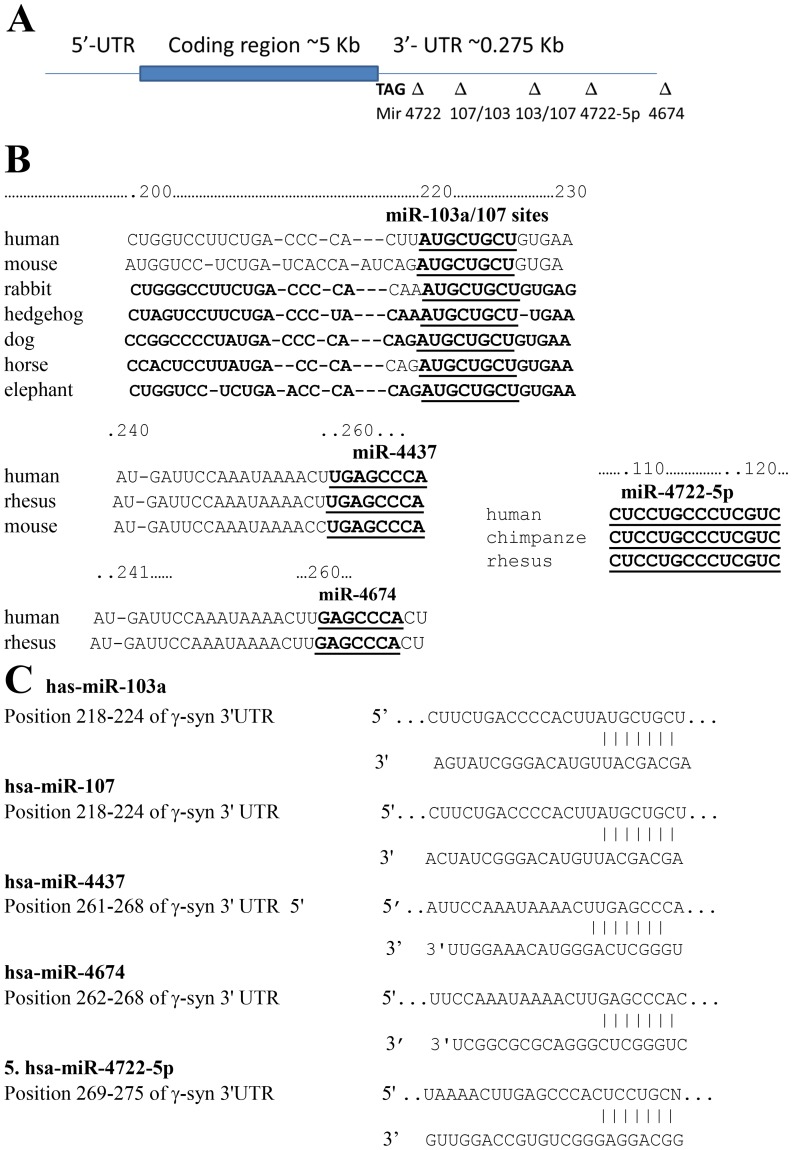
Schematic drawing of γ-synuclein gene and miR targets in 3′-UTR. **A.** γ-Synuclein gene. **B.** Sequence conservation of mir-103a/107, miR-4437, miR-4722-5p and miR-4674 binding sites in γ-synuclein 3′-UTR. **C.** Alignment of mature miR -103a, mir-107, miR-4437, miR-4722-5p and miR-4674 sequences with γ-synuclein 3′-UTR.

Human γ-synuclein gene (GenBank accession no. AF037207) contains a relatively short 3′-UTR (275 bp downstream from stop codon to the polyadenylation site, **Figure 1A**), which produces a single 0.8 kb transcript [13]. To identify putative miRs target sites within γ-synuclein 3′-UTR, predictions from several different algorithms (Target Scan, miRanda, Diana microT, Kertesz algorithm) were compiled and compared. As shown on the Venn diagram (**Figure 1B**), 209 miRs targets were predicted by Kertesz algorithm and 104 using miRNA.org, 55 of them were overlapping. From miRNA-target pairs predicted with Kertesz algorithm (**Table 2**) we have selected the putative miRs target sites which were identified additionally by several prediction algorithms, i.e. miR-103, 107, 424, 497, 4437, 4674, 4722 and analyzed them in detail. The targets for these selected miRs have different level of conservation at orthologous positions across multiple vertebrate species (**Figure 2A and B**). The sequences of complementary regions between these miRs and their targets are shown in **Figure 2C**. A list of miRNA-target pairs with numbers of putative sites and scores is shown in **Table 2**.

**Table 2:** The miRNA-target pairs predicted with Kertesz algorithm (41).

microRNA	Sites	Score*	microRNA	Sites	Score*
hsa-miR-658	4	−22.81	hsa-miR-937	1	−12.44
hsa-miR-608	4	−20.36	hsa-miR-497*	2	−12.4
hsa-miR-744	2	−20.3	hsa-miR-139-3p	1	−12.26
hsa-miR-498	2	−20.06	hsa-miR-601	2	−12.19
hsa-miR-663	3	−19.69	hsa-miR-483-5p	1	−12.15
hsa-miR-486-3p	5	−19.54	hsa-miR-760	1	−11.93
hsa-miR-939	3	−19.05	hsa-miR-195+	2	−11.82
hsa-miR-1207-5p	5	−18.92	hsa-miR-922	4	−11.74
hsa-miR-650	3	−18.66	hsa-miR-449b	1	−11.44
hsa-miR-886-5p	2	−17.75	hsa-miR-449a	1	−11.14
hsa-miR-323-5p	2	−17.69	hsa-miR-661	1	−11.04
hsa-miR-1268*	3	−17.45	hsa-miR-423-5p	2	−10.77
hsa-miR-296-3p	4	−17.21	hsa-miR-638	1	−10.73
hsa-miR-885-3p*	5	−17.18	hsa-miR-663b	1	−10.58
hsa-miR-383	2	−17.09	hsa-miR-877	1	−10.56
hsa-miR-675	1	−17.02	hsa-miR-503+	3	−10.4
hsa-miR-1254	1	−16.46	hsa-miR-34c-5p	1	−10.34
hsa-miR-1293	4	−15.98	hsa-miR-122	3	−10.19
hsa-miR-654-5p	3	−14.96	hsa-miR-1258	1	−10.13
hsa-miR-138*	2	−14.67	hsa-miR-325	1	−10.07
hsa-miR-154	1	−14.66	hsa-miR-659	2	−10.01
hsa-miR-646+	1	−14.61	hsa-miR-485-5p	3	−9.92
hsa-miR-1301	2	−14.24	hsa-miR-520a-5p	1	−9.9
hsa-miR-921	1	−14.01	hsa-miR-525-5p	1	−9.9
hsa-miR-612	3	−13.76	hsa-miR-34a	1	−9.74
hsa-miR-940	3	−13.72	hsa-miR-604	1	−9.58
hsa-miR-103+	4	−13.7	hsa-miR-887	1	−9.51
hsa-miR-298	3	−13.66	hsa-miR-7	1	−9.34
hsa-miR-107+	4	−13.61	hsa-miR-628-3p	1	−9.28
hsa-miR-198	2	−13.45	hsa-miR-493	1	−9.26
hsa-miR-541	5	−13.25	hsa-miR-578	1	−9.18
hsa-miR-611	1	−13.02	hsa-miR-15a+	2	−9.14
hsa-let-7e	2	−12.99	hsa-miR-15b+	2	−9.14
hsa-miR-1321	4	−12.93	hsa-miR-1300	1	−9.1
hsa-miR-665	4	−12.88	hsa-miR-1184	3	−9
hsa-miR-1294	2	−12.73	hsa-miR-765	2	−8.95
hsa-miR-24	1	−12.44	has-miR-424	2	−7.18
hsa-miR-662	1	−12.44			

The energy based score for miRNA–target interaction ΔΔG is shown. The level of miR marked by asterisks * is changed in response to γ-synuclein overexpression.+shows members of the 15/107 family of miRs.

### γ-Synuclein 3′-UTR Alters a Reporter Gene Expression

**Figure 3:**
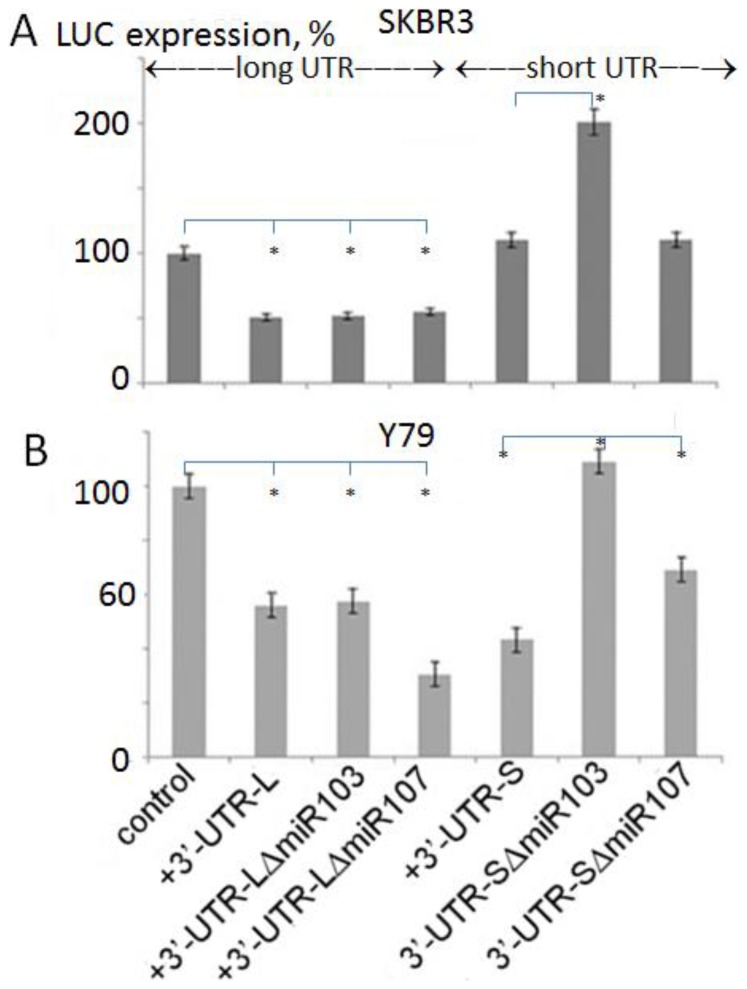
Cell-dependent effect of γ-synuclein 3′-UTR on reporter gene expression. Amount of firefly luciferase activity corrected for renila activity in SKBR3 cells (A) and Y79 (B) following transfection with Luc reporter vector and vector with inserted 3′-UTR. Control – cells were transfected with an empty vector. +3′-UTR-L - cells were transfected with the vector in which a long form of 3′-UTR was inserted downstream of the LUC gene. +3′-UTR-LΔmir-103 - similar to previous, but target for miR-103 was deleted from the UTR; +3′-UTR-LΔmir-107 - similar to previous, but target for miR-107 was deleted. +3′-UTR-S - cells were transfected with the vector in which a short form of 3′-UTR was inserted downstream of the LUC gene. +3′-UTR-SΔmir-103 - similar to previous, but target for miR-103 was deleted from the UTR. +3′-UTR-SΔmir-107 - similar to previous, but target for miR-107 was deleted from the UTR. The blots were repeated three times; stars show statistically significant differences (p<0.05).

To test a functional role of γ-synuclein 3′-UTR we first examined its effect on the efficiency of reporter gene expression. For this purpose the expression of a reporter luciferase (LUC) was measured after transfection of the recombinant construct containing 3′-UTR into cultured cells. Two forms of the 3′-UTR were inserted downstream of the LUC cDNA: a complete form consisting of 275 nucleotides (Long form, L) and a 265 nucleotide form (Short form, S). The effect of both forms on LUC expression was compared with the effect of truncated L and S forms from which targets for several miRs were deleted. The insertion of a long form of γ-synuclein 3′-UTR in the expression vector downstream of LUC gene caused a 51% reduction of LUC activity (+3′-UTR-L) after transfection into SKBR3 (**Figure 3A**) and Y79 cells (**Figure 3B**), confirming the presence of effective regulatory elements in this 3′-UTR fragment.

### Effect of Individual miRs on Gene Regulation

a. Deletion of miR-103 and miR-107. After identifying putative miR targets in γ-synuclein 3′-UTR we examined whether these targets are indeed involved in the regulation of gene expression. Deletion of targets for miR-103 and miR-107 belonging to the miR-15/105 group [42) from the long form (**Figure** **1**) of γ-synuclein 3′-UTR does not cause a significant change in the reporter gene activity (**Figure 3**, 3′-UTR-L-ΔmiR103 and 3′-UTR-L-ΔmiR107), although according to qRT-PCR data these miRs are present in tested cell cultures (not shown). The effect of a short form (UTR-S) from which we have deleted miR-103 and-107 on reporter gene expression is different. The deletion of miR-103 targets from this construct increases LUC activity (**Figure 3** A, B, 3′-UTR-SΔmiR103 and 3′-UTR-SΔmiR-107). Thus, regulatory properties of miR-103 and miR-107 appear only on short form of UTR, from which the targets for miR4674 and miR4722 are deleted (**Figure 1A**). These data suggest the existence of a coordinated mechanism of regulation between these two groups of miRs.

b. miR-4437 and miR-4674 reduce γ-synuclein expression.

**Figure 4:**
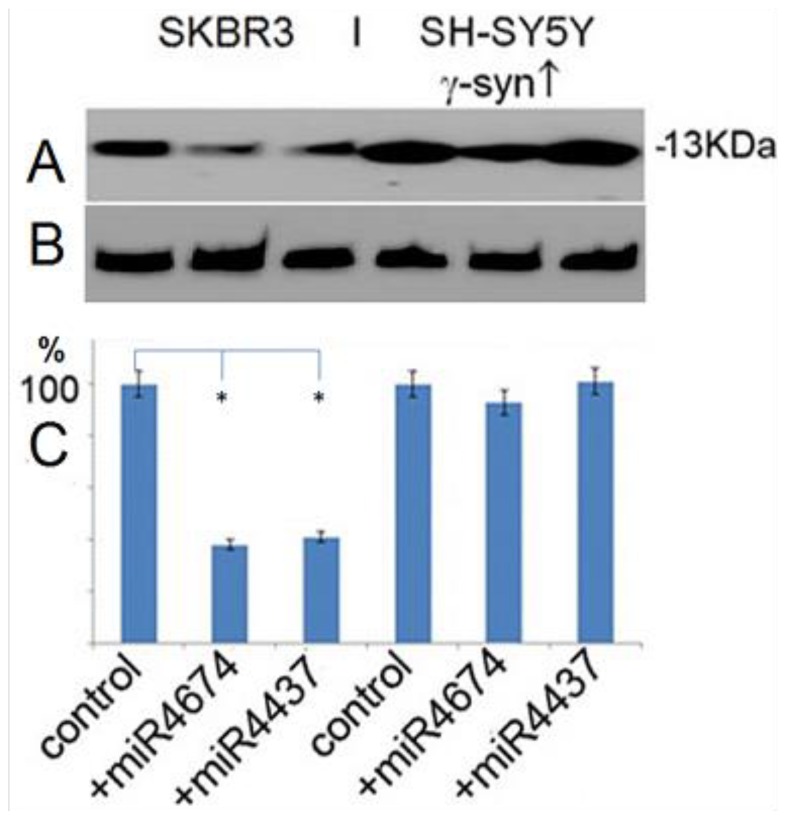
miR-4437 and miR-4674 decrease γ-synuclein expression in SKBR3 with moderate endogenous level of γ-synuclein expression, but do not affect the level of its expression in cells overexpressing γ-synuclein. **A** – Western blotting with antibody to γ-synuclein (Abcam, Cat # ab55424). **B** – Western blotting with antibody to actin (Chemicon MAB1501). **C** - scan of bands shown in **A.** The blots were repeated three times; stars show statistically significant differences (p<0.05).

We then examined whether miR-4437 and miR-4674 for which targets were predicted in the 3′-UTR by bioinformatics search affected γ-synuclein expression in cultured cells. For this purpose we performed expression of these two miRs in SK-BR3 cells using pcDNA 6.2-GW/EmGFP-miR vector. According to the Western blotting data, expression of miR-4674 caused a 61.2% and miR-4437 a 60.1% reduction of endogenous γ-synuclein expression in SKBR3 cells with moderate endogenous level of γ-synuclein expression (**Figure 4**, three left wells). On the other hand, in cells overexpressing γ-synuclein no significant effect of miRs on γ-synuclein expression was found (**Figure 4**, three right wells). These results suggest that miR-4437 and miR-4674 play an important role in the regulation of γ-synuclein expression only in cells with moderate level, but not in cells with elevated level of γ-synuclein.

### γ-synuclein Alters miRNAs

**Figure 5:**
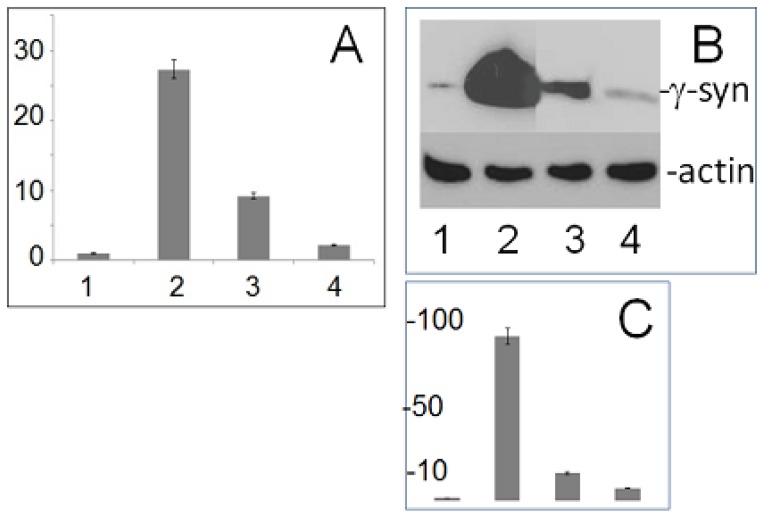
γ-Synuclein mRNA and protein content in four cell lines. **A** - γ-Synuclein mRNA was measured by qRT-PCR with specific primers as described in methods. Relative amount of γ-synuclein mRNA is shown on Y-axis. **B** – Western blotting of cell extracts. The bands were identified with antibody specific to γ-synuclein (upper panel) and actin (bottom panel). **C** – scanning of γ-synuclein specific bands shown in **B**. Extracts from the following cells were loaded on the gel: **1**– SH-SY5Y cells. **2** - SH-SY5Y overexpressing γ-synuclein (B9 clone). **3**– SKBR3 cells. **4**- retinoblastoma Y79 cells. 18S RNA was used as a housekeeping control.

**Figure 6:**
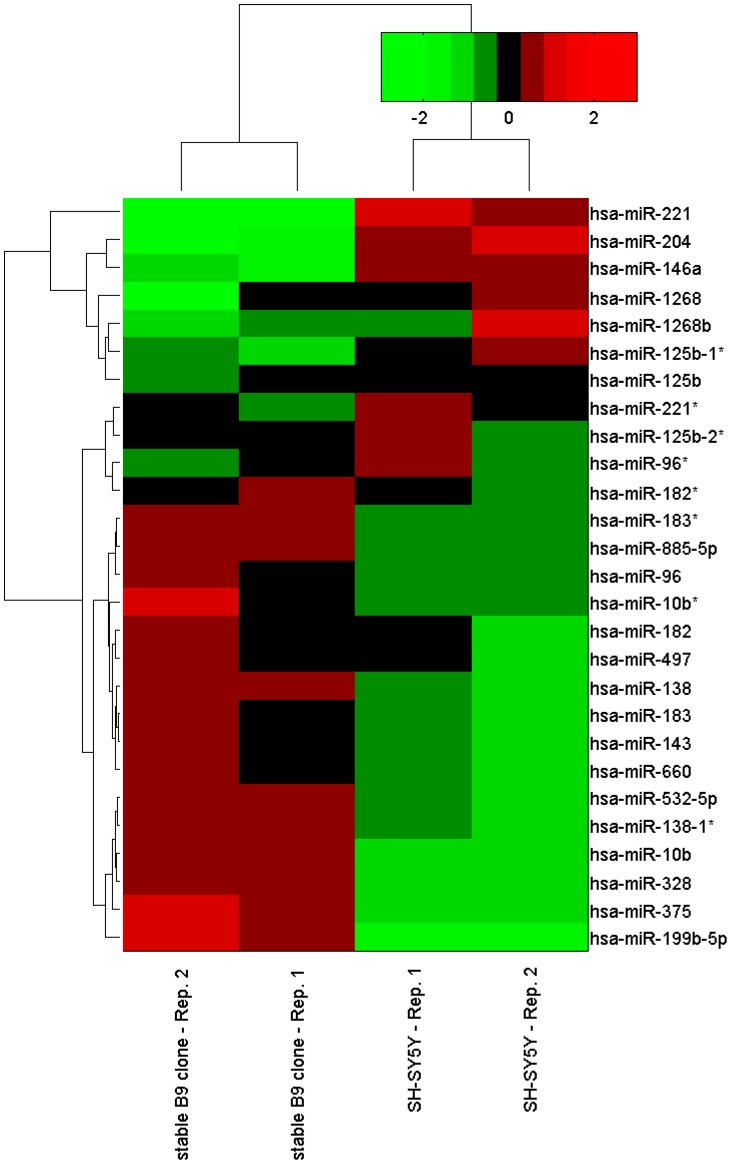
The heat-map shows the distribution of the standardized log expression values of the B9 (γ-synuclein overexpressors) and SHSY5Y (parent strain) samples for the miRs listed in Table 1.

Then we used microarray analysis to determine how the pattern of miRs is changed in response to γ-synuclein overexpression. As shown in **Figure 5**A, in SH-SY5Y cells overexpressing γ-synuclein the level of specific mRNA is elevated approximately 27.4 fold and protein level is 92.1 times higher (**Figure 5**B and C). As shown in **Table 1** and **Figure 6**, γ-synuclein overexpression differentially changes the level of several miRs, upregulating the level of some of them and downregulating the level of others. The most highly upregulated miRs are miR-199b-5p, miR-375 and miR-10b, the most downregulated are miR-221, miR-204 and miR-146a (**Table 1** and **Figure 6**). The data of microarray analysis were further validated by qRT-PCR for miR-199b-5p, miR-328, miR497, miR-885-5p, miR-204 and miR-146a (**Table 1**). Three miRs upregulated as a result of γ-synuclein overexpression, i.e. miR-885-3p, miR-138 and miR-497 are in the list of miRNA-target pairs (**Table 2**). Since all three miRs are elevated in response to γ-synuclein overexpression, they may play an autoregulatory role, reducing γ-synuclein expression. Conversely, miR-1268 which is present in the list of miRs target pairs (**Table 2**) is downregulated by γ-synuclein and also may play an autoregulatory role.

### Biological Functional Analysis

MiRNAs that were significantly differentially expressed (absolute fold-change ≥1.5) between B9 and SH-SY5Y were analyzed by IPA for their association with biological functions. The observed expression patterns of miRNAs let-7e, 10b, 195, 18a, 26b, 126, 132, 145, 410, 183 and 193b indicated a substantial decrease in the activation state (activation z-score −2.4) of biological functions, cellular development and cellular growth and proliferation, associated with the proliferation of carcinoma cell lines. On the other hand the observed expression patterns of the miRNAs, let-7e, 195, 18a, 92b, 143, 145, 146a, 152, 149, 410, 204, 210, 326, 138, 146b-5p, 193a-3p and 483-3p-3p were strongly associated with the increase in the activation state (activation z-score 2.4 - 2.7) of cell death and apoptosis of tumor cell lines. The miRNAs, 10b, 195, 143, 145, 221 were strongly associated with the decrease in the activation state (activation z-score −2.2) of proliferation of vascular smooth muscle cells (Table S1 and Table S2).

### A Crosstalk between γ-synuclein Signaling Pathways and miRNA

**Figure 7:**
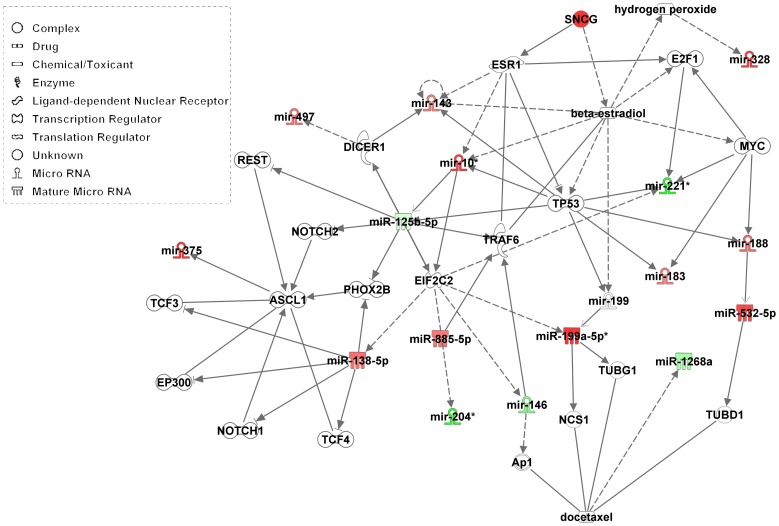
Schematic depiction of the complexity and inter-connectivity of γ-synuclein effect on signaling pathways and miRNA crosstalk. Symbols are used in accordance with the main features of Network Explorer and Canonical Pathways, including molecule shapes and colors as well as relationship labels and types.

A schema of γ-synuclein effect on signaling pathways and miRNA crosstalk is presented in **Figure 7**. γ-Synuclein affects many signaling pathway, transcription factors and the level of miRNAs basically through ligand-dependent nuclear receptor ESR1, beta-estradiol and the tumor suppressor p53. Among the most significant biological functions associated with these molecules are cancer (p-value 1.7E^−10^), cellular development (p-value 1.0E^−09^), cellular growth and proliferation (p-value 1.0E^−09^), cell death and survival (p-value 2.08E^−08^) and cell cycle related biological functions (p-value 4.8E^−08^). In addition, transcription factors E2F1 and ESR1 are involved in the signaling pathways, which are important transcription factors and therapeutic targets in different types of cancer, including BC. These data are in good agreement with previous findings about a substantial role of γ-synuclein in ER-alpha signaling and stimulation of hormone-responsive mammary tumors [7], [8]. Among other transcription factors and transcriptional activators affected by γ-synuclein are activator protein a (AP1), RE1-Silencing Transcription factor (REST), E1A binding protein (EP300), PHOX2B, basic helix-loop-helix transcription factors ASCL1, TCF4 and TCF3 (**Figure 7**). γ-Synuclein acting on these transcription factors and signaling molecules may alter the regulation of many genes, some of which are involved in cell proliferation, and results in the progression of cancer. In addition, γ-synuclein affects centrosome components γ-tubulin (TUBG1) and Δ-tubulin (TUBD1) which are associated with tumorigenesis and tumor progression. Thus the results presented on **Figure 7** and in **Table S1** (“Significant biological functions associated with miRNAs differentially expressed between B9 and SH-SY5Y”) show that the most important biological functions affected by γ-synuclein expression are cancer, cellular development, cellular growth and proliferation, cell death and survival and cell cycle related processes.

## Discussion

Part of the “epigenetic landscape” that shapes oncogenic transformation and robust regulation of gene networks is the miR system controlling both levels and translation of messenger RNA. miRs are key regulators of multiple biological processes including tumorigenesis. Altered expression of miRs is occurs in several tumor types. Here we aimed to understand the association between aberrant γ-synuclein and miRs expression and the advance of cancer and other pathology.

Deregulation of γ-synuclein expression is associated with breast, ovarian and many other forms of cancer [1]–[3], [5], [6] and some neurodegenerative diseases [9]–[13]. Previously transcription regulation and methylation of regulatory regions of the gene have been considered as major regulators of its expression [18]–[20].

Here we show that another mechanism modulates the level of γ-synuclein expression, i.e. miRs mediated posttranscriptional regulation. Furthermore, we used γ-synuclein overexpression as a model to reveal what downstream targets are hit by elevated γ-synuclein level in order to understand what molecular networks are altered in response to its upregulation. The knowledge of these networks is important in order to understand whether γ-synuclein upregulation in pathology is just a passive response to upstream events or its elevated level plays an essential role *per se* switching on downstream pathological mechanisms.

γ-Synuclein gene has a relatively short UTR which contains many putative miRs binding sites predicted by several algorithms (**Figure 1** **and** **2** and **Table 2**). Interestingly, there are 18 putative binding sites for members of the miR-15/107 family of miRs, including miR-15a and -15b, -107, -195, -503 and -646 (**Table 1**). These miRs with 5′-AGCAGC(A) conserved motif which confers specificity have a strong influence on human biology in health and disease [42]. This group contains miRs which act as either tumor suppressors or oncomirs [43]. For example, miR-103/7 which affects reporter gene expression (**Figure 3** **)** and is upregulated both in microarray (**Table 1**) and qRT-PCR analysis (**Figure 5**) promotes metastasis in colorectal cancer [44] and is considered as prognostic biomarker in esophageal carcinoma [45].

The insertion of γ-synuclein 3′-UTR downstream of the reporter gene significantly downregulates the reporter gene expression in two cell types (**Figure 3** **A** and **B,** left part), confirming that it contains miRs binding sites.

The results presented in **Figure 3** indicate that deletion of targets for miR103 and miR107 from a long UTR does not increase reporter gene expression as one could anticipate. Moreover, deletion of miR107 even reduces reporter gene expression in Y79 cells (**Figure 3B**, left part). On the other hand, the deletion of both miR-103 and miR-107 targets from the short UTR significantly increases reporter gene expression in both cell types (**Figure 3**, A and B, right parts). Since from the short form of UTR the targets for miR4674 and 4722 were deleted (**Figure 1A**), we can speculate that there is a coordinated mechanism of regulation operated by these four miRs. According to this putative mechanism, the regulation of expression by miR103 and miR107 is effective only in the absence of targets for miR4674 and 4722, that is when miR4674 and 4722 do not exert their regulatory action. Since the targets for miR4674 and miR4722 are located approximately 40 nucleotides downstream from the targets for mir103 and mir107, this might indicate a coordinated regulation between the effects exerted by these two groups of miRs. The synergistic effect of different miRNAs on the expression level of a single gene has been reported earlier [84].

To further confirm that miRs indeed affects γ-synuclein level in cells we have expressed two of the miRs for which the target sites have been predicted in 3′-UTR, miR-4674 and miR-4437 and analyzed their effect on γ-synuclein expression. In SKBR3 cells with a moderate level of endogenous γ-synuclein expression both miRs caused approximately two-fold reduction of the γ-synuclein level, whereas in cells overexpressing γ-synuclein no inhibitory effect was observed (**Figure 4**). Thus, such gain of function effect of miR-4674 and miR-4437 expression occurs only in cells with physiological level of γ-synuclein expression and is abolished when γ-synuclein is considerably elevated.

Substantial upregulation of γ-synuclein has been found in many types of cancer [1]–[3], [5], [6]. Here we used γ-synuclein overexpression as a model to reveal what miRs and other downstream targets are hit by elevated γ-synuclein level in order to understand what molecular networks are altered in response to elevated level of γ-synuclein. An important aspects of γ-synuclein upregulation is that it interferes with drug-induced apoptotic responses, mediates drug resistance, compromises normal mitotic checkpoint controls, resulting in multi-nucleation and faster cell proliferation [5], [6]. Here we determined for the first time how γ-synuclein overexpression affects miRs.

As we found here, γ-synuclein overexpression significantly alters the level of several miRs (**Figure 5**). It is well established that some miRNAs can serve as either oncogenes or oncosuppressors by targeting oncogenes or oncosuppressors genes [46], [47]. So we asked whether γ-synuclein-induced aberrant regulation of miRs causing their misbalance might have downstream effect by altering intracellular signaling and further promoting cancer phenotype?

Among miRs upregulated by elevated level of γ-synuclein several miRs possess prooncogenic properties, e.g. miR-10b [44]–[46], miR-375 [51], members of the 183/96/182 family [48], [49] and several other miRs (**Table 1**). For example, miR-10b upregulated 3.07 fold in response to γ-synuclein overexpression (**Table 1**) according to published data is associated with tumor metastasis and other clinical characteristics for BC [57]. Furthermore, miR-10b is highly expressed in metastatic BC cells and positively regulates cell migration and invasion. Overexpression of miR-10b in otherwise non-metastatic breast tumors initiates robust invasion and metastasis [48].

miR-375 is upregulated 3.68 fold according to our gene array data (**Table 1**). de Souza Rocha Simonini and coauthors [51] revealed that miR-375 is overexpressed specifically in ERα-positive BC cells. Furthermore, miR-375 is an activator for ERα signaling in BC cells, which promotes cell proliferation. Conversely its inhibition gives rise to an attenuated ERα activity [51]. Interestingly, miR-375 is the first known miR with the capacity to enhance ERα signaling in breast cells and, thus, to promote cell proliferation, whereas all other known miRs with a regulative connection to the ERα pathway act as inhibitors of ERα signaling pathways. miR-199b-5p is highly upregulated in response to γ-synuclein overexpression (**Table 1**). miR-199b-5p expression correlates with metastasis spread in medulloblastoma targeting transcription factor HES1, an effector of the Notch pathway [59].

In cells overexpressing γ-synuclein the level of members of the miR-183/96/182 family is elevated 2.08 times. These miRs are significantly upregulated in patients with lung cancer both in tumor and sera and the elevation is associated with overall poor survival in patients with lung cancer; members of this family are considered as risk factors for lung cancer [52]. Members of this miRs family also plays an oncogenic role in bladder cancer [53]. Since the majority of miRs upregulated in response to γ-synuclein possesses prooncogenic properties, they can increase predisposition to cancer development.

On the other hand, the expression of miR-146 [54], [55] and 125b [50], [56] downregulated by γ-synuclein (**Table 1**) are reduced in BC and some carcinomas, respectively [50]–[52]. Furthermore, miR-221 reported as negative modulators of ERα activity, was found among the most significantly downregulated miRNAs [58] and it is reduced by γ-synuclein overexpression (**Table 1**). A tumor suppressor miR-204 is considerably downregulated in several types of cancers [54].

Therefore, miRs upregulated by γ-synuclein overexpression as shown in **Table 1** and **Figure 6** are increased in several types of cancer and at least some of them have oncogenic properties. Conversely, miRs downregulated by γ-synuclein are usually reduced in cancer and some of them are tumor suppressors. These results suggest that elevation of γ-synuclein level described in BC and other types of cancer might cause a downstream effect which further exacerbates pathological changes characterized for malignancy and might causes more aggressive cancer phenotype.

The mechanism of miR-mediated gene regulation is multifactorial. In *cis*-regulation miRs directly target mRNA and regulate the expression of the target gene at post-transcriptional levels. miRs can also control gene transcription through *trans-*regulation. In this mechanism the effect of miRs is realized indirectly through genes encoding transcription factors, RNA regulating proteins, or genes encoding proteins that interact with the target protein.

Accumulation of aberrant γ-synuclein as a result of its deregulation is described both in neoplasia and some neurodegenerative disorders [9]–[12], [81], affecting its intracellular localization [82] and changing signaling pathways [83]. There is an interesting hypothesis that cancer and neurodegenerative diseases may be influenced by common miRNA pathways that regulate differentiation, proliferation and death of cells. According to this hypothesis, miRNAs act as a central regulator of both oncogenesis and neurodegeneration [23], [60]. Since γ-synuclein is located on the crossroads of these two main human disorders, it is tempting to find what common signaling pathways and miRs it may affect.

Aberrant expression of miR-146a which is downregulated by γ-synuclein (**Table 1**) is implicated in breast cancer [66], [67], Huntington disease [62], Alzheimer’s disease and aged related macular degeneration (AMD) which might be explained by targeting and downregulation of the complement factor H (CFH) [63], [64]. Abnormal regulation of miR-125b which is downregulated by γ-synuclein (**Table** **1**) is associated with breast cancer [56], Alzheimer’s disease [63], [64], [66] and Down syndrome [67]. γ-Synuclein is also associated with glaucoma [11], [12] and its deregulation may play a role in the pathogenesis of this disorder. Therefore we analyzed which miRs changed in response to overexpression of γ-synuclein are present in the retinal ganglion cells (RGC) and other retinal cells, according to previously published data. The following miRs upregulated in response to γ-synuclein are expressed in the retina/RGC: miR-138, 328, 375, 183/96/182 and 497 [61], [65], [80]. On the other hand, miR-221 is downregulated in response to γ-synuclein overexpression is present in the RGC (miRNEye website). These miRs may be considered as candidates mediating γ-synuclein involvement in the glaucomatous alterations. In addition to the effect on multiple miRs, γ-synuclein is able to stimulate the ligand-dependent transcriptional activity of ER-alpha (**Figure 7**), confirming previously published results [7], [8].

In conclusion, this study provided evidence that several miRs regulate γ-synuclein level and set the stage to further evaluate their role in in pathophysiological process. Furthermore, the results presented here demonstrate that miRs provide cell-specific regulation of γ-synuclein expression and set the stage to further evaluate their role in in pathophysiological processes. 3′-UTR contains targets for miRs which are used for the regulation of γ-synuclein expression. This mechanism depends on the level of γ-synuclein and miR expression. An altered level of γ-synuclein expression affects downstream targets [83], including miRs and signaling pathways and might be an important factor in progression of cancer [1]–[6] and some neurodegenerative diseases [11], [85]. Therefore, our data suggest that γ-synuclein is not a mere by-product of upstream events, but an important instigator of malignant progression.

## Supporting Information

Table S1 **Significant biological functions associated with miRNAs differentially expressed between B9 and ShSY5Y.**
(XLSX)

Table S2 (DOC)
