# Supplementary material for: Cell-Specific Post-Transcriptional Regulation of γ-Synuclein Gene by Micro-RNAs
Source: PLoS One. 2013 Sep 11;8(9):e73786. doi: 10.1371/journal.pone.0073786 (PMC3770685; doi:10.1371/journal.pone.0073786)
Supplement: Table S2 — (DOC) [file pone.0073786.s002.doc]

Table S2.

The following primers were used:

**1**. For insertion L and S forms of γ-synuclein 3’-UTR into the vector: Forward (F)-psi-Xho1: 5’-AACTCGAGAGGGCTACAGGCCAG-3’; Reversed (R) psi-Not1: 5’-AAGCGGCCGCGCAGGAGTGGGCTCAAGT-3’; R1apsi-Not1: 5’-AAGCGGCCGCGCTCAAGTTTTATTTGG-3’

**2**. To delete putative targets from γ-synuclein 3’-UTR specific for mir-103 and mir-107 the following set of primers were designed and synthesized: F for deleting mir-103 target:

5’-GGTCCTTCTGACCCCACTTTGTGAATTTTTTTTTTAAATG-3’; Complement primer:

5’-CATTTAAAAAAAAAATTCACAAAGTGGGGTCAGAAGGACC-3’. F primer for deleting a miR-107 target: 5’-CACCCTTGGCCTGTCCAACCAACCTCACTGCCCTC-3’. Complement primer:

5'- GAGGGCAGTGAGGTTGGTTGGACAGGCCAAGGGTG-3’.

**3**. Expression of miRs was carried out using “BLOCK-iT Pol II miR RNAi Expression Vector Kit “(Life Technologies/Invitrogen, Grand Island, NY). The following sets of oligonucleotides were used:

1). hsa-miR-4437. Top strand:

5’-TGCTGTGGGCTCAGGGTACAAAGGTTGTTTTGGCCACTGACTGACTAACCTTTGCCCTGAGCCCA-3’

Bottom strand:

5’-CCTGTGGGCTCAGGGCAAAGGTTGTCAGTCAGTCAGTGGCCAAAACAACCTTTGTACCCTGAGCCCA-3’

2). hsa-mir-4674. Top strand:

5’TGCTGCTGGGCTCGGGACGCGCGGCTGTTTTGGCCACTGACTGACAGCCGCGCCCCGAGCCCAG3’.

Bottom strand:

5’-CCTGCTGGGCTCGGGGCGCGGCTGTCAGTCAGTGGCCAAAACAGCCGCGCGTCCCGAGCCCAG-3’

3). hsa-mir-103

Top strand:

5’-TGCTGAGCAGCATTGTACAGGGCTATGTTTTGGCCACTGACTGACATAGCCCTACAATGCTGCT-3’

Bottom strand:

5’-CCTGAGCAGCATTGTAGGGCTATGTCAGTCAGTGGCCAAAACATAGCCCTGTACAATGCTGCT-3’.
